# How Children Become Sensitive to the Morphological Structure of the Words That They Read

**DOI:** 10.3389/fpsyg.2017.01469

**Published:** 2017-09-05

**Authors:** S. H. Deacon, Kathryn A. Francis

**Affiliations:** ^1^Department of Psychology and Neuroscience, Dalhousie University, Halifax NS, Canada; ^2^Nova Scotia Hearing and Speech Centres Halifax, NS, Canada

**Keywords:** morphology, derived words, reading, elementary school, base frequency, family size, family frequency

## Abstract

**Background:** We tested the predictions of models of word reading development as to the effects of repeated exposure on reading of derived words.

**Aim:** Our goal was to examine the impacts of variables that quantify different aspects of this exposure: base frequency, family frequency, and family size.

**Methods and Samples:** In Experiment 1, we asked 75 children in Grades 3 and 5 to read derived words with low surface frequencies (e.g., *questionable*) that varied in base frequency, family frequency, and family size. In Experiment 2, we asked 41 adults to read the same set of words.

**Results:** In Experiment 1, only base frequency made a contribution to word reading accuracy that was independent of the other two variables of interest (family size and family frequency) and the control variables (surface frequency, semantic relatedness, and neighborhood size). In Experiment 2, a similar pattern of results emerged, this time on reading speed.

**Conclusion:** Together, results of these two studies suggest that base frequency has a special role in both children’s and adults’ reading of derived words. These findings suggest that it plays a specific role in development and maintenance of sensitivity to morphological structure in reading.

## Introduction

The English language encodes the minimal units of sound (i.e., phonemes) and of meaning (i.e., morphemes) in the spelling of words (Chomsky and Halle, 1968). A large body of research demonstrates that an appreciation of the phonological basis of print is central to young children’s reading development (e.g., Share, 1995; National Reading Panel, 2000; Ehri, 2005). To support their reading, children are likely to benefit from an appreciation of the morphological basis of words in print, at least by the upper elementary school period. Over 40% of the unfamiliar words that Grade 5 students encounter in texts are derived from more frequent words (Nagy et al., 1993). Derived words are morphologically complex forms that typically involve a change in word class from their base forms; for example, the adverb *seriously* is derived from the more frequent adjectival base *serious*. There is clear evidence that children are sensitive to the morphological structure of derived words in their reading (e.g., Mann and Singson, 2003). This is demonstrated, for example, through increased accuracy or speed in reading derived words than control words (e.g., *hilly* vs. *silly*, the former of which can be divided into the morphemes *hill* + *y;* Carlisle and Stone, 2005). Deacon et al. (2011) found that Grades 4 and 6 children were more accurate and faster in reading derived words with high than with low base frequencies. We present here the results of an investigation designed to add precision to theoretical models of the development of sensitivity to morphological structure of words in reading.

Models of the development of both reading and lexical representation point to a key role of repeated exposure to morphemes in the establishment of sensitivity to morphological structure of words. As an example, Ehri (2005) suggests that repeated exposure to the letter-patterns that make up morphemes allow children to consolidate these units in their reading. Similarly, Schreuder and Baayen (1995) suggest that the representation of morphologically complex words emerges through monitoring the developing lexicon for connections between form and meaning. The implication of form in this model of lexical representation suggests that it could apply equally to reading as to lexical representation. The processes described in both of these models predict a role of repeated exposure to morphemes and morphologically complex words in establishing sensitivity to the morphological structure of words.

These predictions of effects of repeated exposure leave us wondering which aspects of exposure impact children’s sensitivity to the morphological structure of words, especially as it relates to reading. It could be that repeated exposure to the diversity in form across multiple members of the same morphological family (e.g., *prediction*, *predictable*, *predictability*…) enables development of sensitivity to the morphological structure of these words in reading. Family size is a count of the number of morphological relatives of a base, while family frequency is the summed frequency of those morphological relatives (e.g., Taft, 1979). Of the two, family frequency likely more closely approximates frequency of exposure to all members of the morphological family. The variety across family members might be key to the extraction of letter-patterns that represent morphemes (Ehri, 1995) and to the detection of form and meaning co-occurrences (Schreuder and Baayen, 1995). Testing the effects of family size and family frequency would determine whether exposure to the diversity in morphological family members plays a role in the establishment of sensitivity to morphological structure in reading.

Alternatively, development of sensitivity to morphological structure in reading might be influenced by exposure to isolated instances of the base form, such as *predict*. Exposure to the base on its own might be the most potent form of repeated exposure to morphemic letter-patterns (e.g., Ehri, 2005) and to form and meaning co-occurrences (e.g., Schreuder and Baayen, 1995). If this is the case, we would expect the frequency of the base to play a privileged role in children’s reading of derived words. Testing these theoretically driven predictions requires an empirical study designed to separate the effects of base frequency, family size and family frequency on derived word reading.

Multiple studies show impacts of base frequency on English-speaking children’s reading of derived words. Mann and Singson (2003) found that third and sixth Grade children were more accurate in reading derived words with high than with low base frequencies. Similar effects emerged in Deacon et al.’s (2011) study of children in Grades 4, 6, and 8, particularly with low frequency derived forms. Similar effects emerged in Carlisle and Stone’s (2005) study of Grades 4 and 5 children, although no such effects emerged in Grades 2 and 3. These studies present initial evidence of effects of base frequency on children’s reading of derived words, at least by the middle of the elementary school years; notably, none of these studies controlled for or investigate effects of either family frequency or family size.

Turning to these other variables, Carlisle and Katz (2006) showed effects of both family frequency and family size on children’s reading of derived words. Grades 4 and 6 English-speaking children were faster and more accurate in reading sets of words with high than with low family frequencies. These word sets also differed in base frequency and family size. The children were also faster and more accurate in reading words with large than small families (see also Perdijk et al., 2012 in Dutch). These word sets also differed in base and derived word frequency. Carlisle and Katz’s further analyses showed strong relationships between base frequency and family frequency, as well as between family size and surface frequency. As such, Carlisle and Katz urge caution in interpreting effects uncovered in their study. As an example, they note that “because the small and large families differed in base word frequency, interpretation of the results of this analysis is not clear-cut” (p. 684). Studies of children’s reading have not yet isolated the effects of base frequency, family size, and family frequency, which is key to specifying models.

Recent studies of adult lexical representation have made clear progress in disentangling these effects. Ford et al. (2010) used correlational analyses at the item level to investigate the continuous, rather than binary, effects of variables. With this approach, Ford et al. (2010) found that family size and base frequency each had an independent effect on lexical decision response times (see also Burani and Caramazza, 1987). Family frequency counts were not included in these analyses. This evidence of independent effects of family size is consistent with that of other studies. In contrast, independent effects of base frequency have not always emerged in such studies (Schreuder and Baayen, 1997; Bertram et al., 2000). The analytic approach taken by Ford et al. (2010) in studies of adult lexical representation could be useful in isolating the aspects of repeated exposure on sensitivity to morphological structure of words in reading.

The current study was designed to provide the requisite empirical data to specify the aspects of repeated exposure to morphologically complex words that determine child and adult sensitivity to the morphological structure of words in reading. We used a correlational design to evaluate the independent effects of each of base frequency, family size and family frequency on derived word reading in a manner that controls for their likely inter-correlation (e.g., Carlisle and Katz, 2006). In two separate studies, we evaluate the effects of base frequency, family size and family frequency, beyond control variables, on children’s (Experiment 1) and adults’ (Experiment 2) reading of low frequency derived words. We focus on low frequency derived forms given evidence of morphemic effects particularly on these forms (e.g., Carlisle and Stone, 2005).

We predict that exposure to the base on its own is required to establish sensitivity to morphological structure in word reading, as reflected in independent effects of base frequency. While it is possible that children are able to extract morphological structure across family members, we suspect that repeated exposure to precisely the same form is required to support the detection of shared form and meaning (e.g., Schreuder and Baayen, 1997). In testing such a prediction, it is critical to statistically control for the possibility that effects of base frequency are due to sheer repetition of the orthographic or phonological form, without attention to morphology. As such, we control for orthographic neighborhood size to limit the influence of orthographic familiarity effects that are not specific to either the base form or morphological relatives (see e.g., Perdijk et al., 2012). This also effectively controls for phonological influences. Further, and following on Ford et al. (2010), we include controls for semantic relatedness in order to disentangle morphemic from semantic effects (see also Schreuder and Baayen, 1997; De Jong et al., 2000). We also include controls for surface frequency given the demonstrated impacts of frequency on children’s and adults’ reading (Spieler and Balota, 1997; Spencer, 2010).

## Experiment 1

In the first experiment, we tested the influence of base frequency, family size, and family frequency on children’s accuracy in reading derived words. We recruited children in Grades 3 and 5, an age at which they should be sensitive to the morphemic structure of written words (Ehri, 2005). Mid to late elementary years is also a period during which children are learning rapidly about the many derived words both in print and in oral language (Carlisle, 1988; Anglin, 1993; Nagy et al., 1993; Carlisle, 2000). Finally, Grades 3 and 5 are years in which we expect children to be able to manage the task demands of reading derived words out loud. Examining the independent influence of these three variables in children’s reading of derived words will specify the aspects of repeated exposure that support the development of children’s sensitivity to the morphemic structure of derived words in their reading.

### Method

#### Participants

We recruited 43 children in Grades 3 and 32 in Grade 5 from two rural schools on the east coast of North America. Mean age of the children was 8;11 years (*SD* = 3.9 months) in Grade 3 and 10;11 years (*SD* = 4.2 months) in Grade 5. All children had parental consent and parents reported children as speaking English as a first language. All children were enrolled in standard English curriculum. We have no reason to believe that any were bilingual. The children read within the normal range for their age (standard scores within 85 to 115 on standardized tasks); mean standard word reading scores (Woodcock, 1998) were 100.12 (*SD* = 12.49) and 93.06 (*SD* = 10.84) in Grades 3 and 5, respectively.

#### Materials

We selected 48 derived words based on children’s word frequencies (Zeno, 1995; see Appendix). All target words had a low surface frequency that ranged from 1 to 3 words/million words across the 12 grade levels in the corpus (U). To ensure developmental appropriateness, all words ended in one of 6 suffixes (-*ment*, -*ness*, -*ly*, -*ful*, *-able*, and -*less*). All of these were rated one of the 20 most common suffixes (Blevins, 2001) and they were all highly productive. There were no phonological or orthographic changes between the base and derived forms (e.g., *adapt*-*adaptable*), as both variables are known to affect children’s processing (e.g., Carlisle, 1988).

The 48 words varied in family size, family frequency, and base frequency. Following on prior research with children, all inflected, derived, and compound family members of the base were included in calculations of family size and family frequency (Carlisle and Katz, 2006). We also calculated these values a second time in which we included only derived and compound family members (following on Ford et al., 2010). We obtained Latent Semantic Analysis semantic relatedness values for the base-derived word pairs (Landauer et al., 1998); these correlate highly with subjective ratings of semantic relatedness (Rastle et al., 2000). To control for orthographic familiarity that is not specific to the base or morphological relatives, we calculated neighborhood sizes for the base and derived forms. Words of the same length with a letter substitution at one position were considered to be neighbors (e.g., Coltheart et al., 1977).

#### Procedure

We asked the children to read the 48 words as part of a larger battery of tasks administered individually to students in a quiet area of their schools. In this derived word reading task, each word was presented on a computer screen using DirectRT (Jarvis, 2000). Children saw a fixation point on the screen for one second. Next, each target word appeared on a black screen in white 40 point Arial font. Each word was presented once and disappeared 500 ms after the child read it aloud. Words were displayed for a maximum of 8000 ms. Children read three practice words prior to reading the set of experimental items. The experimenter recorded children’s reading accuracy.

### Results

Across both grades, children read an average of 55% (*SD* = 24%) of words correctly (51 and 61% in Grades 3 and 5, respectively). Reliability for accuracy was high (alpha = 0.89). Analyses focused on accuracy. Accuracy was deemed appropriate in this study given that the scores obtained from the children clearly demonstrate that their ability to read these words is still developing (e.g., Carlisle and Katz, 2006). Interpretable analyses of speed rely on high levels of accuracy.

#### Correlations

In all correlational and linear regression analyses, we report analyses in text and tables with calculations of family size and family frequency with all inflected, derived, and compound family members. This is the standard approach in prior studies with children (e.g., Carlisle and Katz, 2006). We confirm these results in analyses with calculations of family size and family frequency that include only derived and compound family members; this is standard approach in prior studies with adults; Ford et al., 2010).

Pearson correlations across items are shown in **Table 1**. These are calculated on a by-item basis to determine how word features, such as base frequency and family size, are related within words. Base frequency was highly correlated with both family frequency and family size, and family frequency and family size are also significantly correlated with one another. All correlations between experimental variables were below 0.70, ensuring that there were no multicollinearity concerns (Tabachnick and Fidell, 2007).

**Table 1:** Correlations between measures of family size, base frequency, family frequency, surface frequency, semantic relatedness, neighborhood size of the derived word, and neighborhood size of the base word.

Variable	Family	Base	Family	Surface	Semantic	Neighborhood	Neighborhood
	size	frequency	frequency	frequency	relatedness	size (derived)	size (base)
Family size	1	0.337^∗^	0.663^∗∗∗^	0.027	0.102	-0.017	0.211
Base frequency	–	1	0.574^∗∗∗^	0.033	0.058	-0.046	0.128
Family frequency	–	–	1	-0.174	-0.037	-0.163	0.143
Surface frequency	–	–	–	1	0.005	0.149	0.352^∗^
Semantic relatedness	–	–	–	–	1	0.110	-0.178
Neighborhood size (derived)	–	–	–	–	–	1	0.335^∗^
Neighborhood size (base)	–	–	–	–	–	–	1

*^∗^*p* < 0.05, ^∗∗^*p* < 0.01, ^∗∗∗^*p* < 0.001. Correlations with family size and family frequency counts are reported for values calculated with all compound, derived, and inflected members (Carlisle and Katz, 2006).*

#### Linear Regression Analyses

We conducted linear regression analyses to determine if base frequency, family size, and family frequency each contributed to children’s derived word reading accuracy. These analyses with mean accuracy for each derived word (following on Carlisle and Katz, 2006) were conducted on a by-items basis (following on Ford et al., 2010).

We examined data for missing values and distribution normality (Tabachnick and Fidell, 2007). The one missing value was corrected with missing value replacement. Skew in base frequency, family frequency, family size, and surface frequency was corrected with square root, log, square root, and inverse transformations, respectively. Analyses below are reported with the transformed data; analyses with raw data revealed the same pattern of results.

The linear regression analyses are reported in **Table 2**. In these analyses, we included surface frequency, semantic relatedness, and neighborhood size of the base and derived forms as the first step in each equation (following on Ford et al., 2010). In three separate linear regression analyses, we rotated the order of entry of family size, base frequency, and family frequency at each of Steps 2, 3, and 4. Results at Step 2 show the effects of each of these variables after the control variables only. Each of base frequency, family frequency, and family size made a significant contribution to children’s accuracy in reading derived words, after our control variables. Results at Step 4 evaluate the unique effects of each of base frequency, family frequency, and family size. Only base frequency emerged as a unique predictor (at 8.3%) of children’s derived word reading accuracy, beyond the substantial control variables.

**Table 2:** Summary of linear regression analyses predicting word accuracy for children in Grades 3 and 5.

Step	Predictor	*B*	Δ*R*^2^	Predictor	*B*	Δ*R*^2^	Predictor	*B*	Δ*R*^2^
1	Surface frequency	-0.152	0.241^∗^						
	Semantic relatedness	0.204							
	*N* size of base	0.537							
	*N* size of derived form	-0.050							
2	Family size	0.206	0.039	Base frequency	0.376	0.137^∗^	Family frequency	0.275	0.066ˆ
3	Family frequency	0.241	0.028	Family size	0.099	0.008	Base frequency	0.337	0.073^∗^
4	Base frequency	0.349	0.077^∗^	Family frequency	-0.004	0.000	Family size	0.101	0.005


*ˆp < 0.1, ^∗^p < 0.05; reported Beta weights are Step Betas. N, neighborhood. Values at Step 2 show the effects of each of family size, base frequency and family frequency after controlling for surface frequency, semantic relatedness and the neighborhood size of the base word and its derived form. Values at Step 4 show the effects of each of family size, base frequency and family frequency after controlling for surface frequency and semantic relatedness, in addition to the other two variables*.

We computed interaction terms with grade (Pedhazur and Schmelkin, 1991) to evaluate possible differences in effects across our two grades. Each interaction term was included as the fifth step in a separate linear regression, following on the four-step regressions described above. None of the interaction terms were significant; beta values ranged from -0.079 to -0.049, *t’*s from -0.647 to -0.400, and all *p*’s > 0.519. The absence of interactions suggests that the pattern of findings did not differ by grade.

We confirmed all linear regressions analyses with family size and family frequency variables calculated with only derived and compound family members (as is standard in adult research; Schreuder and Baayen, 1997; Ford et al., 2010). Again, base frequency emerged as the only unique predictor of children’s derived word reading accuracy. As before, this contribution survived controls for surface frequency, semantic relatedness, family frequency and family size.

### Discussion

Our first experiment investigated the unique influences of base frequency, family size and family frequency on Grades 3 and 5 children’s derived word reading accuracy. Our analyses addressed the interrelationships between these variables (Carlisle and Katz, 2006). Our results suggest that only base frequency had a unique impact on derived word reading accuracy, beyond the influence of the others. These results were consistent across our two groups of late elementary school children, as reflected in the absence of interactions. These results were also consistent across different metrics of family size and family frequency (e.g., Schreuder and Baayen, 1997; Carlisle and Katz, 2006) and beyond multiple control variables.

## Experiment 2

The results of Experiment 1 with children suggest a unique influence of base frequency on children’s derived word reading accuracy beyond that of family size and family frequency. This independent influence of base frequency is similar to that uncovered in Ford et al.’s (2010) study with adults. This comparability in results across studies suggests developmental stability. Nevertheless, it would be useful to confirm the pattern of results with adults in precisely the same paradigm—that of word reading, rather than lexical decision. Further, it would be important to do so with controls for both family size and family frequency, the latter of which was omitted from Ford et al.’s (2010) analyses. This was the goal of our second experiment.

### Method

#### Participants

We recruited 41 undergraduate students at a large research-intensive university on the east coast of North America. All had English as a first language. The mean age of the participants was 21;00 years (*SD* = 2;05 years).

#### Materials

The materials were identical to those in Experiment 1.

#### Procedure

Participants were tested in a quiet room in the Psychology department; all other aspects of the procedure were identical to those in Experiment 1.

### Results

Across participants, 99% (*SD* = 2%) of words were read correctly. Given this high level of accuracy, analyses focused on word reading speed. Correlational analyses of item features (such as base frequency and family size) are the same as for Experiment 1 (see **Table 1**), given that the same items are included. As in Experiment 1, there were no concerns with multicollinearity (Tabachnick and Fidell, 2007).

#### Linear Regression Analyses

Analyses were conducted by-items, with the mean speed of reading for each derived word was the focus of the analyses. The data was examined for missing values and distribution normality. As with the children’s data, skew in base frequency, family frequency, family size, and surface frequency variables was corrected with square root, log, square root, and inverse transformations, respectively. Analyses are reported with the transformed data; analyses with raw data revealed the same pattern of results.

Three linear regression analyses examined whether each of base frequency, family size, and family frequency made a significant contribution to adults’ speed of derived word reading, after controlling for surface frequency and semantic relatedness. Accordingly, surface frequency, semantic relatedness, and neighborhood size of the base and derived forms were entered as the first step in the equation. These analyses are reported in **Table 3**. As before, each of family size, base frequency, and family frequency were rotated in Steps 2, 3, and 4 in the regression analyses. Results at Step 2 show that only base frequency made a significant contribution to adults’ speed of reading derived words, beyond semantic relatedness, surface frequency, and neighborhood size of the base and derived forms. Results at Step 4 show that the unique effects of each of base frequency, family size and family frequency. Only base frequency emerged as a unique predictor (at 9.3%) of adults’ speed of derived word reading.

**Table 3:** Summary of linear regression analyses predicting word reading speed for adults.

Step	Predictor	*B*	Δ*R*^2^	Predictor	*B*	Δ*R*^2^	Predictor	*B*	Δ*R*^2^
1	Surface frequency	0.217	0.166ˆ						
	Semantic relatedness	-0.242							
	*N* size of base	-0.415							
	*N* size of derived form	0.054							
2	Family size	-0.143	0.019	Base frequency	-0.335	0.108^∗^	Family frequency	-0.171	0.026
3	Family frequency	-0.132	0.009	Family size	-0.043	0.002	Base frequency	-0.360	0.084^∗^
4	Base frequency	-0.373	0.088^∗^	Family frequency	0.129	0.006	Family size	-0.111	0.006


*ˆp < 0.1, ^∗^p < 0.05; reported Beta weights are Step Betas. N, neighborhood. Values at Step 2 show the effects of each of family size, base frequency and family frequency after controlling for surface frequency, semantic relatedness and the neighborhood size of the base word and its derived form. Values at Step 4 show the effects of each of family size, base frequency and family frequency after controlling for surface frequency and semantic relatedness, in addition to the other two variables*.

The same pattern of results emerged when linear regressions were conducted with family frequency and family size counts that included only derived and compound family members (following on prior adult research, Schreuder and Baayen, 1997; Ford et al., 2010).

### Discussion

Our second experiment investigated the influence of base frequency, family size and family frequency on adults’ reading of derived words. As in Experiment 1 with children, only base frequency had a unique influence beyond all others; these effects emerged in Experiment 2 on the dependent variable of speed of reading. These results confirm similarity in the unique influence of base frequency across age levels (adults vs. children) and across measurement type (speed vs. accuracy).

## General Discussion

Current models of reading development (e.g., Ehri, 2005) propose a role for repeated exposure to morphemes in the establishment of sensitivity to morphemic information in children’s reading. Our work was designed to specify the impacts of this morphemic exposure. We did so by extending prior investigations into the effects of a single morphemic variable (e.g., base frequency in Deacon et al., 2011) and evaluating the unique effects of each of family size, family frequency, and base frequency on reading of derived words. Our experimental approach included controls for surface frequency and semantic relatedness (e.g., Ford et al., 2010), as well as neighborhood size (Perdijk et al., 2012). Experiment 1 examined these effects on Grades 3 and 5 children’s derived word reading accuracy and Experiment 2 investigated these effects on adults’ derived word reading speed. In both experiments, only base frequency had a unique impact on derived word reading that survived our multiple controls, including family size and family frequency.

Our finding of an independent effect of base frequency on children’s accuracy in derived word reading extends prior studies. Several prior studies with factorial designs have demonstrated effects of base frequency on children’s reading of derived words (Mann and Singson, 2003; Carlisle and Stone, 2005). These did not, however, control for family size and family frequency. Amongst others, Carlisle and Katz (2006) noted that there is clear overlap in the relationships between these variables, and pointed to the need to investigate their independent effects on children’s reading of derived words. The results of Experiment 1 show robust effects of base frequency on children’s derived word reading accuracy that survive controls for family size and family frequency, in addition to surface frequency, semantic relatedness, and neighborhood size. These results emerge across Grades 3 and 5 and across the two different metrics of family size and frequency (e.g., Carlisle and Katz, 2006; Ford et al., 2010).

Unique effects of base frequency on derived word reading also emerged in our study of adults’ reading speed. As in the data with children, these effects emerged following controls for family size and family frequency, in addition to semantic relatedness, surface frequency, and neighborhood size. The parallel results across Experiments 1 and 2 suggest consistency in the effects of base frequency in the development and consolidation of morphemic effects on derived word reading. The emergence of these effects in accuracy in children and in speed with adults is in keeping with the age level at which we measured these effects. The mid-range accuracy levels for the Grade 3 and 5 children (roughly 55%) shows that skill in reading these words is clearly still developing. In contrast, the high rates of accuracy for the adults (roughly 99%) suggest that these participants had likely reached some degree of automaticity in reading these words. Despite differences in the metric in which they emerge, the consistency of results point to stability in the factors affecting both developing and skilled derived word reading.

Our findings of the unique effects of base frequency on adults’ derived word reading extend prior research. There is a good deal of related research on lexical representation. In such a study, Ford et al. (2010) demonstrated effects of base frequency, following controls for semantic relatedness, surface frequency and family size, in adults’ lexical decisions for derived words. Our results show that these base frequency effects survive an additional control for family frequency, at least when tested in a word reading paradigm. That said, our results are perhaps surprising given the findings of other prior studies demonstrating impacts of family size independently of base frequency and family frequency on adult lexical decision (e.g., Schreuder and Baayen, 1997). To apply these results to understanding of lexical representation, it would be important to replicate our study in a lexical decision paradigm. Our results suggest that there are clear effects of base frequency on adults’ speed in reading derived words, suggesting effects on adults’ representations as they are applied to word reading.

These findings have clear theoretical implications. Prominent developmental models have highlighted the importance of repeated exposure to morphemes (Schreuder and Baayen, 1995; Ehri, 2005). In the Introduction, we outlined the two most plausible alternative ways in which repeated exposure to morphemes might influence the development of children’s sensitivity to morphological structure in derived word reading. Repeated exposure might occur across members of the same morphological family (as would be reflected in family size and/or family frequency effects) or through exposure to an isolated base form (as would be reflected in a base frequency effect). We found that only the effects of base frequency were independent of others, both in child and adult readers. These unique effects of base frequency on children’s reading suggest that isolated exposure to a base word is particularly influential for the development of sensitivity to morphemic structure in reading. Critically, these effects emerged beyond controls for neighborhood size; neighborhood size of the base reflects any likely influences of exposure to simple orthographic patterns. As such, remaining effects are likely to be due to exposure to base morphemes, rather than simple letter-patterns. Consistency in these effects in adults suggests that this exposure to isolated bases is also important in the maintenance of this sensitivity. Consistent exposure to base forms on their own appears central to the detection and retention of form-meaning connections.

These findings also have clear educational implications. It perhaps seems intuitive that teaching children about morphemes should occur across the presentation of multiple related family members. Our data show, however, that children do not appear to pick up on these commonalities, at least not in an implicit manner. Children might need explicit teaching about connections in meaning across morphological families, perhaps with special attention to the shared base. Of course, such educational implications need empirical testing.

We also need to interpret our results in accordance with their limitations. One lies in the cross-sectional nature of our study. Our results can speak to the factors that influence derived word reading at each of Grades 3 and 5 and in adulthood, but it cannot address directly the factors that influence development across these years. We believe that the consistent pattern of findings across these age levels suggests consistency in the factors that influence change over time; a longitudinal study is needed to confirm this suggestion. Another limitation lies in the nature of the items that we tested. Our conclusions apply specifically to low frequency derived words with no phonological or orthographic changes between base and derived forms. It would be important to extend this investigation to other word types. Finally, in our focus on item-level factors, this experiment did not assess possible child level factors; a much larger sample would be needed to do so. Possible variables for investigation include vocabulary and word reading (see e.g., Levesque et al., 2017).

## Conclusion

Our results suggest a unique role for base frequency in the establishment and maintenance of sensitivity to morphemic information in derived word reading. Models have specified repeated exposure as central to the establishment of morphemic sensitivity in reading (e.g., Ehri, 2005). The results from our two experiments suggest that it is exposure to the base on its own supports the development of morphemic sensitivity in children’s derived word reading accuracy and its maintenance in adults’ speed of reading.

## Ethics Statement

The research protocol was approved by the Social Sciences and Humanities Research Ethics Board of Dalhousie University. Child assent and parental consent was obtained for all participating children, in addition to consent for all participating adults.

## Author Contributions

SHD led the writing of the manuscript. KF led data analysis.

## Conflict of Interest Statement

The authors declare that the research was conducted in the absence of any commercial or financial relationships that could be construed as a potential conflict of interest.
