# Supplementary material for: How Children Become Sensitive to the Morphological Structure of the Words That They Read
Source: Front Psychol. 2017 Sep 5;8:1469. doi: 10.3389/fpsyg.2017.01469 (PMC5591820; doi:10.3389/fpsyg.2017.01469)
Supplement: Supplementary file 1 [file Data_Sheet_1.pdf]

Appendix

List of Derived Words Used in Reading Complex Words and their U Frequencies According to Zeno Word Frequency Guide (1995).

Derived word	Base Word	Family Frequency	Family Size	Base Frequency	Surface Frequency	Semantic Relatedness	N Size of Derived Word	N Size of Base Word
adaptable	adapt	34.22	25	9	1	.33	1	3
adjustable	adjust	46.07	18	14	1	.57	0	0
alignment	align	3.46	13	0.75	1	.56	0	1
ceaseless	cease	14.41	8	3	1	.21	0	4
cleverness	clever	2	2	18	1	.34	0	2
confinement	confine	12.13	5	1	1	.10	0	1
cowardly	coward	2.66	3	1	1	.29	1	3
cruelly	cruel	4.04	4	13	1	.46	3	2
dampness	damp	2.73	12	22	1	.26	0	10
densely	dense	21.46	11	16	1	.30	1	3
diligently	diligent	1	1	1	1	.31	0	0
engagement	engage	27.92	10	8	2	.22	0	1
falsely	false	2.63	10	21	1	.43	0	0
fateful	fate	1.63	4	12	1	.37	2	20
fondness	fond	2.75	5	13	1	.35	0	11
forgetful	forget	8.5	7	58	1	.28	0	5
forgiveness	forgive	2.95	7	5	1	.30	0	1
frightful	fright	57.32	13	4	2	.30	0	4
generously	generous	1.07	3	9	1	.29	0	0
gentleness	gentle	70.61	16	33	1	.43	0	2
hateful	hate	25.13	9	26	1	.32	1	23
locally	local	4.9	12	107	1	.29	2	4
mournful	mourn	6.54	8	1	1	.22	0	0

Derived word	Base Word	Family Frequency	Family Size	Base Frequency	Surface Frequency	Semantic Relatedness	N Size of Derived Word	N Size of Base Word
narrowly	narrow	10.23	6	77	1	.11	0	7
nourishment	nourish	5.98	11	0.9	3	.44	0	0
peculiarly	peculiar	2.45	4	14	1	.34	0	0
purposeful	purpose	40.99	11	113	1	.30	0	0
questionable	question	201.52	13	176	2	.13	0	0
relentless	relent	2.62	5	0.05	1	.03	0	4
resentful	resent	9.84	9	1	1	.10	0	4
respectful	respect	36.65	19	47	2	.40	0	1
richness	rich	29.76	12	122	1	.33	0	10
roughness	rough	17.05	28	49	1	.44	1	9
senseless	sense	49.24	7	145	1	.17	0	4
seriousness	serious	25.02	3	84	2	.42	0	0
shyness	shy	3.11	5	13	1	.49	1	14
slowness	slow	230.73	9	88	1	.34	0	17
smoothness	smooth	23.02	10	57	1	.53	0	1
soberly	sober	1.66	5	3	1	.05	0	6
sorrowful	sorrow	3.03	5	7	1	.60	0	3
sparsely	sparse	2.02	2	1	2	.34	0	0
stainless	stain	8.28	12	3	1	.11	0	7
stubbornly	stubborn	2.43	2	5	2	.35	0	0
suspiciously	suspicious	2.08	3	7	2	.24	1	2
truthful	truth	4.62	8	61	1	.29	0	1
uniqueness	unique	2	2	26	1	.67	0	0
urgently	urgent	1	1	7	1	.27	1	0
weightless	weight	21.35	22	112	1	.41	0	3

Notes. N = Neighbourhood
